# Immune Checkpoint Inhibition for Pancreatic Ductal Adenocarcinoma: Current Limitations and Future Options

**DOI:** 10.3389/fimmu.2018.01878

**Published:** 2018-08-15

**Authors:** Derya Kabacaoglu, Katrin J. Ciecielski, Dietrich A. Ruess, Hana Algül

**Affiliations:** Internal Medicine II, Klinikum rechts der Isar, Technische Universität München, Munich, Germany

**Keywords:** pancreatic ductal adenocarcinoma, immune checkpoint inhibitors, triple E, antigenicity, immunogenicity, tumor microenvironment

## Abstract

Pancreatic ductal adenocarcinoma (PDAC), as the most frequent form of pancreatic malignancy, still is associated with a dismal prognosis. Due to its late detection, most patients are ineligible for surgery, and chemotherapeutic options are limited. Tumor heterogeneity and a characteristic structure with crosstalk between the cancer/malignant cells and an abundant tumor microenvironment (TME) make PDAC a very challenging puzzle to solve. Thus far, targeted therapies have failed to substantially improve the overall survival of PDAC patients. Immune checkpoint inhibition, as an emerging therapeutic option in cancer treatment, shows promising results in different solid tumor types and hematological malignancies. However, PDAC does not respond well to immune checkpoint inhibitors anti-programmed cell death protein 1 (PD-1) or anti-cytotoxic T lymphocyte-associated antigen 4 (CTLA-4) alone or in combination. PDAC with its immune-privileged nature, starting from the early pre-neoplastic state, appears to escape from the antitumor immune response unlike other neoplastic entities. Different mechanisms how cancer cells achieve immune-privileged status have been hypothesized. Among them are decreased antigenicity and impaired immunogenicity *via* both cancer cell-intrinsic mechanisms and an augmented immunosuppressive TME. Here, we seek to shed light on the recent advances in both bench and bedside investigation of immunotherapeutic options for PDAC. Furthermore, we aim to compile recent data about how PDAC adopts immune escape mechanisms, and how these mechanisms might be exploited therapeutically in combination with immune checkpoint inhibitors, such as PD-1 or CTLA-4 antibodies.

## Introduction

Pancreatic ductal adenocarcinoma (PDAC), as one of the most fatal malignancies in the world, is the fourth leading cause of cancer-related deaths among both men and women in developed countries (1). Its mortality almost equals its incidence: for 2018 alone, 55,440 diagnoses of pancreatic cancer are projected for the United States with 44,330 associated deaths in the same year (2). At the time of diagnosis, only a minority of patients have localized, resectable tumors (10%); while most patients display locally advanced disease (29%) and/or distant metastasis (52%), and the remainder are not even staged (2). The 5-year survival rate of pancreatic cancer is only around 7–8% in the United States, which is likely due to late stage diagnosis (2, 3). The high number of estimated pancreatic cancer-related deaths can be hypothesized to be due to several factors: first, late and unspecific symptoms, as well as a lack of PDAC-specific markers or screening resources result in late diagnosis at an advanced stage. Second, delayed diagnosis leads to low resection rates, since most of the tumor patients present with locally advanced or metastatic disease. And third, PDAC is characterized by a low response to radiotherapy (RT) and chemotherapy, which, at least in part, is due to its dense desmoplastic stroma impairing drug delivery (4–6). Also, targeted therapies including small drug inhibitors of key molecular signaling pathways associated with PDAC progression showed almost none (i.e., MEK and PI3K) or only mild benefits (e.g., EGFR) with a moderate increase in overall survival (7–12). Recent advances in chemotherapeutic options for PDAC appear to provide a survival benefit that will likely not be sufficient to decrease mortality rates (13–15). Hence, in developed countries, PDAC is expected to be the second leading cause of cancer-related deaths by 2030 (1, 16). Impaired efficacy of chemotherapy or targeted therapies in cancer was associated with innate and acquired resistance through genetic and epigenetic instability of cancer cells (17, 18). Immunotherapy offers great potential for the treatment of tumors displaying such resistance. Especially T cells with their ability to generate receptors recognizing the heterogeneous and specific repertoire of tumor-related antigens provide great promise in cancer therapeutics. The adaptive immune response might even form an immunological memory providing long-term tumor control. Studies revealing how T cells function under pathophysiological conditions led to development of immune checkpoint inhibitors, which have been successfully translated into the clinic. Thus far, immune checkpoint inhibition (ICI) has shown promising results for the treatment of solid tumors, including melanomas (19–22), as well as lung (23–27), renal (28, 29), bladder (30–32), and head and neck cancers (33, 34), as well as in hematological malignancies, such as Hodgkin’s disease (35, 36) and follicular or diffuse-large B-cell lymphoma (37). Although single-agent treatment with immune checkpoint inhibitors showed great promise with many solid tumors, their effect on PDAC has been quite disappointing (38, 39).

Here, we want to discuss the unique characteristics of PDAC immune evasion and why PDAC is unresponsive toward checkpoint inhibition. First, we will provide details concerning immune checkpoint inhibitors and their mechanism of action. Second, the immune-privileged nature of PDAC will be examined. Then, the antigenic and immunogenic attributes of PDAC and how tumor cell-intrinsic and -extrinsic factors within the tumor microenvironment (TME) regulate immunogenicity will be comprehensively discussed, including options for pharmacological modulation of these mechanisms to increase ICI therapy response in the clinic.

## Immune Checkpoint Inhibitors

T cells with their various subsets are involved in the regulation of immune responses in autoimmune diseases, but also against infections and cancer. In TME or in tumor-resident lymph nodes, professional antigen-presenting cells (APCs) such as dendritic cells (DCs) display tumor-specific antigens to naïve T cells through major histocompatibility complexes (MHCs) in a process called priming (40). Antigen presentation through MHC-class II acts on naïve CD4^+^ T cells, giving rise to Th_1_, Th_2_, and Foxp3^+^ regulatory T cell (T_reg_) subtypes, which are all important for immune response orchestration (41): Th_1_ polarization induces cytokines (characterized mainly by IFNγ production) further augmenting MHC expression in APCs (42) and antitumor T cell and macrophage cytotoxic activity (43). Th_2_ polarized cells are characterized by IL-4 and IL-13 production, leading to exhaustion of T cells and enhancement of other tumor-promoting responses (44, 45). T_regs_ get activated once the effector T cell activation reaches a threshold. With the release of immunosuppressive cytokines (TGF_β_ and IL-10) T_regs_ negatively regulate T cell effector function (46). On the other hand, antigen presentation through MHC-class I leads to differentiation of naïve CD8^+^ T cells into cytotoxic T lymphocytes (CTLs), which are directly able to kill antigen-expressing cancer cells (41). Upon MHC:antigen engagement, activated T cells clonally expand in secondary lymphoid organs, and traffic into the inflammatory sites to execute their functions and release intermediary cytokines and ligands to provoke helper immune cells for further support (40).

T cell-mediated immune response is tightly regulated *via* both the repertoire of immunosuppressive cells in the microenvironment and cell-intrinsic regulation of anergy and exhaustion (47). T cell anergy is the state of T cells in which they are hyporesponsive to triggers of naïve T cell differentiation (47). And T cell exhaustion describes a process by which effector T cells become resistant to persistent reactivation (47). Under physiological conditions, T cell activation upon MHC engagement is balanced *via* co-regulation of both stimulatory and inhibitory signals, referred to as immune checkpoints. The balance between stimulatory and inhibitory signals is crucial to generate self-tolerance and to maintain the ability to fight with non-self. However, tumor cells shift this balance toward their benefit by abrogating co-activatory signals and augmenting co-inhibitory signals ultimately heightening anergy and exhaustion (48).

Cytotoxic T lymphocyte-associated antigen 4 (CTLA-4 or CD152) and programmed cell death protein 1 (PD-1 or CD279) are the most studied co-inhibitory receptors of T cell receptor (TCR) signaling (40). The first antibody against CTLA-4, ipilimumab, was approved in 2011 (19), while pembrolizumab and nivolumab, antibodies that both target PD-1, were approved in 2014 for the treatment of melanoma (20, 21, 38). The clinical success of antibodies targeting CTLA-4 and PD-1 marks a breakthrough as these agents established immunotherapy as a new pillar of cancer treatment strategies next to surgery, chemotherapy, and radiation therapy (49).

After TCR engagement with cognate peptide presented by a MHC molecule, costimulatory receptor CD28 binding with CD80 (B7.1) or CD86 (B7.2) amplifies TCR signaling (50). CTLA-4, on the other hand, has higher affinity for CD80 and CD86, outcompeting CD28 binding (50, 51), and subsequently sequestering CD80 and CD86 from the APC surface (52). Initial TCR activation with CD28 co-activation increases IL-2 release, which induces metabolism, proliferation, and survival in a paracrine manner. However, gradual CTLA-4 accumulation on the T cell membrane replaces the activation signal of CD28, blocking IL-2 accumulation (53). Since B7 proteins are expressed on APCs but not on solid tumor cells, the action of CTLA-4 inhibition is thought to take place in secondary lymphoid organs where early T cell activation occurs. CTLA-4 action on CD8^+^ CTLs is inhibitory, as shown in several studies (54, 55). Still, the overall inhibitory action of CTLA-4 is thought to mainly show itself through its action on CD4^+^ Foxp3^+^ T_regs_, indirectly modulating CD8^+^ CTL action (48). T_regs_ produce CTLA-4 constitutively through the action of their subset defining transcription factor Foxp3 (56–58). Deletion of CTLA-4 in T_regs_ reduces their activity, blocking their immune-suppressive action (59, 60). Still, use of CTLA4 antibodies in preclinical mouse models of PDAC did not affect T_reg_ infiltration in tumors while enhancing total CD4^+^ T cell presence (61). T_regs_ might also mediate effector T cell activation through APCs, impairing their B7 ligand expression, and thereby decreasing the CD28 co-activation signal on effector T cells (52). Overall, CTLA-4 engagement downregulates effector T cell activity, while enhancing T_reg_ immunosuppressive activity (59, 62). Inhibiting CTLA-4 action might enhance immunosurveillance through both its action on effector and T_regs_.

Programmed cell death protein 1 belongs to the family of CD28 proteins, initiating co-inhibitory signaling upon TCR engagement (63, 64). Ligands of PD-1 receptor PD-L1 (B7-H1 or CD274) and PD-L2 (B7-DC or CD273) belong to the B7 family of proteins (64–67). PD-1 is expressed mostly on late effector phase CD4^+^ helper T cells and CD8^+^ cytotoxic T cells in peripheral tissues (63, 68). Especially chronically activated, then exhausted CD8^+^ cytotoxic T cells show constitutive PD-1 production (69–72). Therefore, PD-1 action is mostly associated with the late phase of immune response, which counterbalances cytotoxic T cell activity. PD-1 is also expressed on T_regs_ and PD-1 blockage leads T_reg_ apoptosis (73). Also, PD-L1 stimulation of naïve T cells can skew differentiation toward the T_reg_ subset (74). Therefore, anti-PD-1 treatment might show an indirect effect on antitumor T cells through its inhibitory actions on T_regs_ (75).

Programmed cell death protein 1 knock out mice show reduced peripheral tolerance and display autoimmunity (76, 77), with a milder phenotype compared with CTLA-4 knock out mice (78, 79). There is a prominent difference between CTLA-4 and PD-1 effects. Anti-CTLA-4 action mostly results in changes in secondary lymphoid organs during the initial phase of naïve T cell activation, while anti-PD-1 treatment targets the effector phase of T cell activation in the periphery where the activated T cells attack the target (40, 48, 80). In addition, CTLA-4 is mobilized to the cell membrane upon TCR engagement in naïve T cells directly from the protein stores, implicating its importance for initial T cell activation (81). By contrast, PD-1 transport requires an initial transcriptional production causing a 6–12 h delay in response upon TCR engagement (48). Considering the differences in mode of action between CTLA-4 and PD-1, PD-1 blockage is thought to be effective in TME (80). Tumor-infiltrating lymphocytes (TILs) in the TME are frequently exhausted due to chronic exposure to the tumor antigens and PD-L1 directly produced by the tumor cells or anti-inflammatory cells of the TME (82). Anti-PD-1 or anti-PD-L1 therapy aims to reduce this exhausted state of TILs in the TME. Of note, PD-1 blockage (e.g., nivolumab) shows milder autoimmunity-related side effects than anti-CTLA-4 treatment (e.g., ipilimumab) in melanoma patients (19, 83). Considering anti-CTLA-4 and PD-1 therapy has implications in different phases of immune response, combination therapy with nivolumab and ipilimumab showed prolonged progression free survival and a higher objective response rate than ipilimumab alone, albeit with concomitant higher toxicity (84).

## The Immune-Privileged Nature of PDAC: Immunosurveillance and Immunoediting

**Figure 1:**
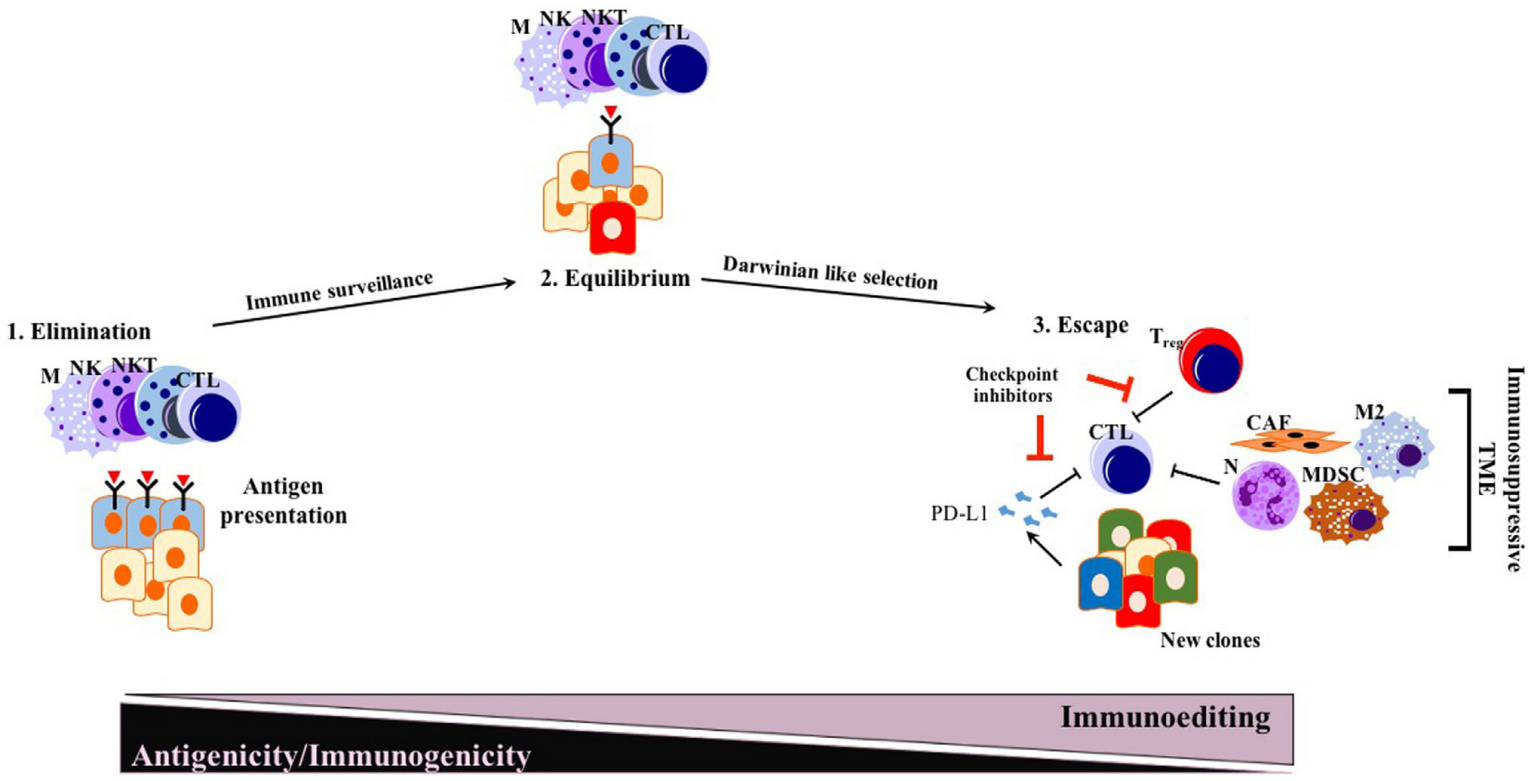
Conventional triple E hypothesis: elimination, equilibrium, and escape. While many solid tumors responding to ICI therapy follow triple E of immunoediting, PDAC is mostly an exception. Generated new clones due to Darwinian-like selection reduce their antigenicity and immunogenicity, escaping from immunosurveillance. Abbreviations: M, macrophages; NK, natural killer; NKT, natural killer T cells; CTLs, cytotoxic T lymphocytes; N, neutrophils; M2, M2 phenotype macrophages; MDSCs, myeloid-derived suppressor cells; T_reg_, regulatory T cells; CAFs, cancer-associated fibroblasts; ICI, immune checkpoint inhibition.

The immunosurveillance hypothesis was proposed by Paul Ehrlich (85) in the early 1900s and later developed further by Thomas and Burnet (86, 87). As a very important concept for cancer immunotherapy, immunosurveillance states that immune cells continually survey somatic cells for any malignant transformation to then destroy them (88). The concept of cancer immunoediting is a byproduct of the immunosurveillance process, in which cancer cells undergo a Darwinian-like selection for their capacity to evade an attack by the immune system (88). The concept of tumor immunoediting proposed by Schreiber and colleagues in 2002 states three different phases of tumor immunoediting: elimination, equilibrium, and escape (i.e., triple E hypothesis) (Figure 1) (88, 89). As being more comprehensive than immunosurveillance, immunoediting proposes that not only innate immunity but also adaptive immunity is involved in the elimination process of tumor cells. During the equilibrium phase, tumor cell variants surviving the dynamic but relentless pressure of adaptive and innate immunity undergo a Darwinian-like selection. At the end of the equilibrium phase, many of the tumor cells are dead, whereas new clones generated, likely through genetic instability with better resistance to the immune response, remain. In the escape phase, survivors of the equilibrium phase start to expand in numbers, maintaining an immune-privileged state (89).

**Figure 2:**
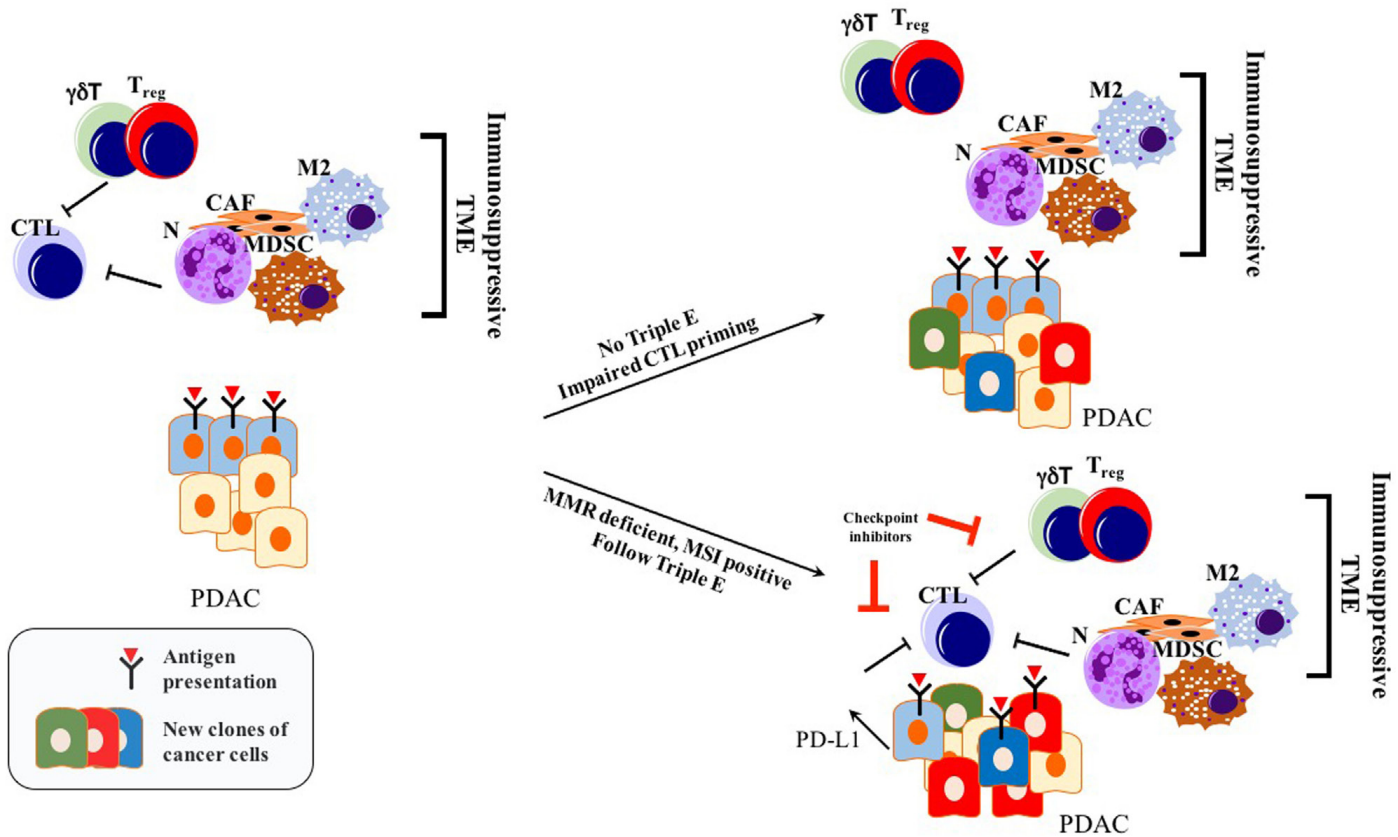
Immunoediting in PDAC: only tumors with genetic instability follow Triple E, while others cannot. Immunosuppressive TME blocks initial CTL priming. Therefore, cancer cells are not forced to undergo Darwinian-like selection. PDAC can still retain its antigenic capacity while impairing immunogenicity making it unresponsive to checkpoint inhibitors. Abbreviations: CTLs, cytotoxic T lymphocytes; N, neutrophils; M2, M2 phenotype macrophages; MDSCs, myeloid-derived suppressor cells; T_regs_, regulatory T cells; CAFs, cancer-associated fibroblasts; γδT, γδT cells; MMR, mismatch repair; MSI, microsatellite instability; TME, tumor microenvironment.

Before the wide use of genetically engineered mouse models (GEMMs) of PDAC, human or mouse tumor transplantation into mice had been the main model for preclinical studies of therapeutic response (90). To eliminate simple tissue rejection of tumor xenografts, mostly immune-incompetent mouse models had been utilized. However, these models are unsuitable for studies of the immune response toward tumors. Furthermore, syngeneic murine transplantation models do not provide information regarding the tumorigenesis process. GEMMs for PDAC harboring pancreas-specific expression of mutant Kras recapitulate carcinogenesis of human PDAC, as pre-neoplastic lesions (PanIN) reliably progress to invasive and metastatic cancer (91). In this mouse model, CD45^+^ leukocytes were shown to accumulate in time as the disease progresses. However, CD4^+^ T cells observed in PanIN lesions were mostly of the Foxp3^+^ T_reg_ subtype, accompanied by an abundance of myeloid-derived suppressor cells (MDSCs) and M2 macrophages (92). Strikingly, infiltration by CD8^+^ antitumor cytotoxic T cells was very scarce in early PanIN lesions, and only a small portion of advanced tumors actually showed presence of active CD8^+^ CTLs (92). This spontaneous carcinogenesis model of PDAC highlights the immune-privileged status of PDAC even in the early neoplastic state (92). Unlike for many other solid tumors, the elimination phase of the triple E hypothesis is almost absent or substantially impaired during murine carcinogenesis due to the scarcity of cytotoxic immune cells and the abundant presence of immunosuppressive cells (92). Thus, ablation of T cells did not affect the spontaneous formation of cancer in KPC models (LSL—Kras^G12D/+^; LSL—Trp53^R172H/+^; Pdx—1Cre) (93). However, ectopic expression of a strong neoantigen (e.g., ovalbumin) in cancer cells boosted T cell-mediated immunity, rescuing the elimination phase of the immunoediting sequence. Expression of a single, yet strong, neoantigen thus allowed tumor control *via* CTL infiltration and “Triple E” (immune active) immunoediting. This implies that the scarcity of neoantigens in PDAC is not a result of the elimination step of immunoediting, but rather due to an alternative mechanism more like immune quiescence (Figure 2). Because of immune quiescence in tumors with low basal adaptive immune activation, CTLs cannot invade into the TME to initiate conventional immunoediting during carcinogenesis, which is true for the KPC model (93). This model represents human PDAC fairly well, showing an “immune quiescence like” phenotype rather than an “immune active” one (94). Reduced CD8^+^ CTL and increased CD4^+^ Foxp3^+^ T_reg_ infiltration in progressive PDAC has also been validated in human patient samples (95).

In summary, PDAC frequently does not undergo a Darwinian-like selection with respect to the adaptive immune response. Thus, it retains vulnerability toward the natural T cell repertoire. Thus, strategies boosting T cell priming, activation levels, and attraction are promising for the treatment of this cancer (96).

## Factors Determining the Efficacy of ICI and Failure in PDAC

**Figure 3:**
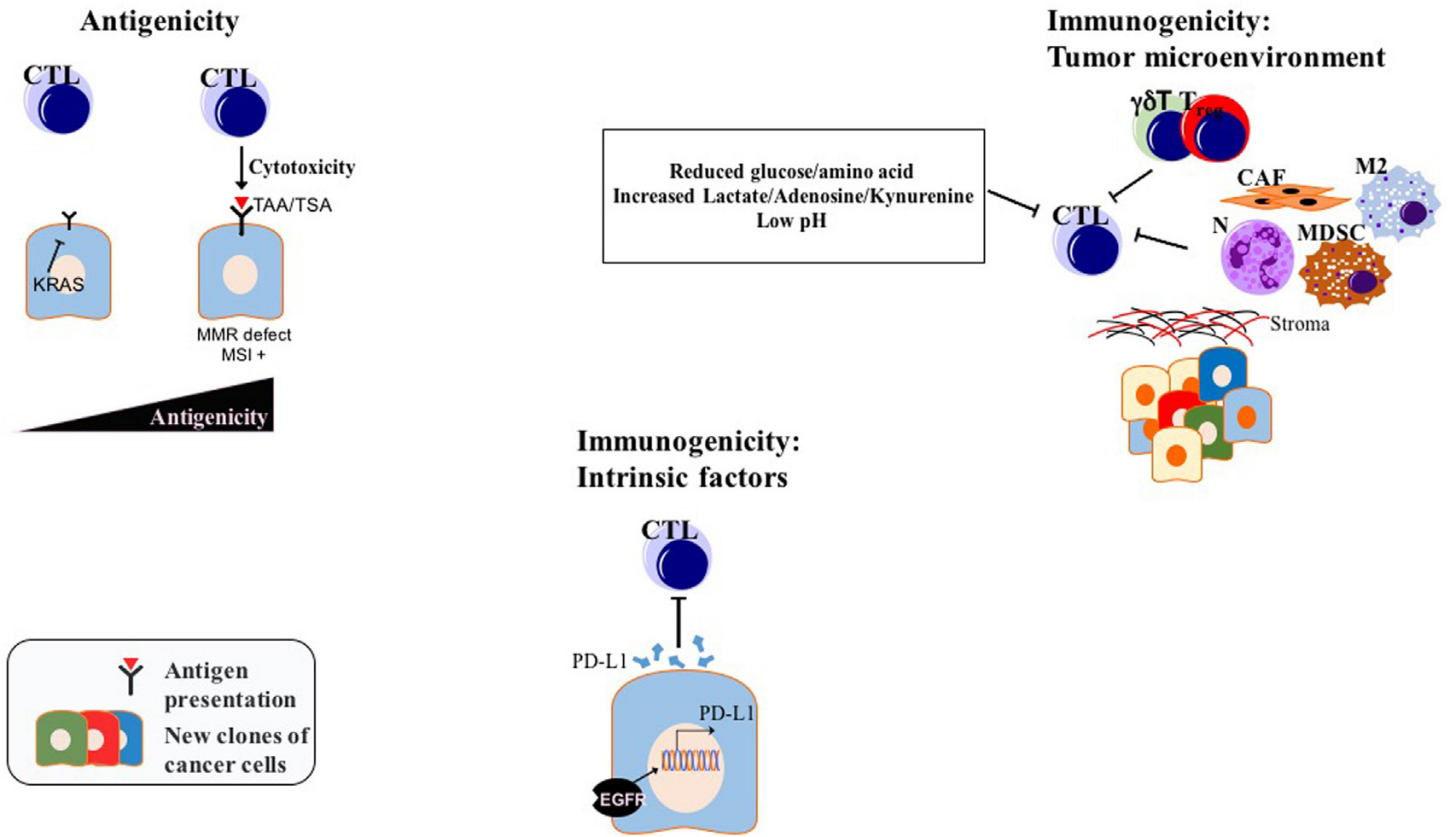
Factors determining ICI efficiency in PDAC: while modulation of antigenicity, intrinsic immunogenicity, and extrinsic immunogenicity *via* TME might be valid for many tumors, drawn examples above are experimentally shown for PDAC. Abbreviations: TAAs, tumor-associated antigens; TSAs, tumor-specific antigens; CTLs, cytotoxic T lymphocytes; N, neutrophils; M2, M2 phenotype macrophages; MDSCs, myeloid-derived suppressor cells; T_regs_, regulatory T cells; CAFs, cancer-associated fibroblasts; γδT, γδT cells; MMR, mismatch repair; MSI, microsatellite instability; TME, tumor microenvironment; ICI, immune checkpoint inhibition.

Two important factors determine the prospects of immunotherapy of cancer in general, and checkpoint inhibition, in particular, antigenicity and immunogenicity, the latter being modulated by both intrinsic properties of tumor cells and TME (97) (Figure 3). Considering the, to date, low efficacy of immunotherapy, and especially checkpoint inhibition in PDAC, a better understanding of the immune escape mechanisms present in PDAC will pave the way for combination factors of checkpoint inhibition for the treatment of this generally intractable disease. A list of selected preclinical mouse model studies focusing on ICI combination therapies in PDAC can be found in Table 1.

**Table 1:** Selection of studies focusing on immune checkpoint inhibition (ICI) combination therapies in preclinical mouse PDAC model.

Combination approach	Method	Preclinical mouse model	Control group/treatment	Experimental group/treatment	Results	Reference
Oncogenic signaling	MEK inhibition	Subcutaneous transplantation of KP^lox/+^C mouse cell line	Either MEKi (GSK1120212) or mPD-1-Ab	MEKi and mPD-1-Ab	Reduced tumor growth and possible regression	(140)

Stromal remodeling	FAP^+^ cell depletion	KP^R172H^C transgenic mouse model with modified *fap* gene driving diphtheria toxin receptor expression in FAP^+^ cell	Only diphtheria toxin (DTx)	DTx with mPD-L1-Ab	Reduced tumor volume	(158)
		
			Only diphtheria toxin (DTx)	DTx with cytotoxic T lymphocyte-associated antigen 4 (CTLA-4)-Ab	Deceleration of tumor growth	
	
	CXCR4 inhibition	KP^R172H^C autochthonous mouse model	CXCR4i (AMD3100) with isotype control	CXCR4i and CTLA-4-Ab	No effect
			CXCR4i (AMD3100) with isotype control	CXCR4i and mPD-1-Ab	Reduced tumor growth

	Focal adhesion kinase (FAK) inhibition	Syngeneic and orthotopic tumor transplantation of mouse PDAC cell lines isolated from KP^lox/+^C mice	Low dose gemcitabine with either FAKi (VS4718) or mPD-1-Ab	Low dose gemcitabine with FAKi and mPD-1-Ab	Reduced tumor burden, improved overall survival	(160)
			Low dose gemcitabine with either FAKi or anti-CTLA4	Low dose gemcitabine with FAKi, and CTLA-4-Ab	No benefit	
			Low dose gemcitabine with FAKi and mPD-1-Ab	Low dose gemcitabine with FAKi and mPD-1-Ab and CTLA-4-Ab	Reduced tumor burden	
		KP^lox/lox^C autochthonous mouse model	Low dose gemcitabine with mPD-1-Ab and CTLA-4-Ab	Low dose gemcitabine with FAKi and mPD-1-Ab and CTLA-4-Ab	Increased survival, 2/15 mice are long-term survivors	
	
	Interleukin 6 (IL-6) targeting	Isolated cancer cells from KP^R172H^C mice and Pan02 cells were subcutaneously transplanted, KPC-luc cells orthotopically transplanted into C57BL/6 mice	Either isotype control or anti-IL-6 or mPD-1-Ab	Anti-IL-6 and mPD-1-Ab in combination	Reduced tumor growth	(169)
		KPC-Brca2 autochthonous mouse model	Isotype control	Anti-IL-6 and mPD-1-Ab in combination	Extended overall survival	
	Hyaluronan depletion	Orthotopic transplanted KP^R172H^C-luc cells or KPC-Brca autochtonous mice	Either *Salmonella*-based sh-IDO (shIDO-ST) delivery or PEGPH20	*Salmonella*-based sh-IDO (shIDO-ST) delivery and PEGPH20	Reduced tumor burden, increased overall survival	(177)

Myeloid compartment	Cluster of differentiation 40 (CD40) agonist	Subcutaneously transplanted KP^R172H^C cells	Either gemcitabine/nab-paclitaxel or CD40 agonist-Ab	Gemcitabine/nab-paclitaxel and CD40 agonist-Ab	Higher tumor regression, enhanced survival, reduced overall tumor growth rate, maintained T cell memory	(203)

	CXCR2 inhibitors	KP^R172H^C autochthonous mouse model	mPD-1-Ab treatment with vehicle	mPD-1-Ab treatment with CXCR2 SM (AZ13381758)	Extended survival, 2/14 mice long-term survivors	(212)

	CSF1R inhibitors	Orthotopic transplantation of KC-INK4A/Arf^lox/lox^	Gemcitabine with either vehicle or CTLA-4-Ab or CSF1Ri (PLX3397)	Gemcitabine with CTLA-4-Ab and CSF1Ri	More than 90% reduced tumor progression	(216)
			Either vehicle or CTLA-4-Ab and mPD-1-Ab combination, or CSF1Ri	CTLA-4-Ab, mPD-1-Ab, and CSF1Ri combination	Completely blocked tumor progression, 15% tumor regression	
			Gemcitabine with either vehicle or CTLA-4-Ab and mPD-1-Ab combination, or CSF1R-Ab	Gemcitabine with CTLA-4-Ab, mPD-1-Ab, and CSF1R-Ab combination	Completely blocked tumor progression, 85% tumor regression	

Metabolic regulation	Glucocorticoid treatment	Pre-cachectic KP^R172H^C autochthonous	Isotype and PBS treatment	CXCR4i (AMD3100) with mPD-L1-Ab	Arrested PDA growth	(254)
			Isotype, PBS, and corticosterone treatment	CXCR4i (AMD3100), mPD-L1-Ab, and corticosterone	PDA is no more arrested, tumor growth in control and experimental groups was same	
	
Radiotherapy	Radiation with ICI	Subcutaneous transplantation of KP^R172H^C cell line	Either treatment of CTLA-4-Ab or mPD-1-Ab or radiation, or dual combinations	CTLA-4-Ab, mPD-1-Ab, and radiation triple combination	Extended survival	(269)
	Radiation with CD40 agonist-Ab	Subcutaneous and orthotopic transplantation of KP^R172H^C cell line	Radiation with CTLA-4-Ab and mPD-1-Ab	Radiation, CTLA-4-Ab, mPD-1-Ab, and CD40 agonist-Ab	Increased abscopal effect, extended survival	(270)

### Antigenicity

Antigenicity refers to the ability of tumor cells to produce and present tumor-specific antigens (TSA) and tumor-associated antigens (TAAs) to the adaptive immune system (97). The bottlenecks of antigenicity include the range of TAA and TSA production, and their ability to be presented to the immune system through MHC complexes (human leukocyte antigen—HLA—in humans) (97, 98). TAAs are overexpressed in cancer cells while their expression is low in normal cells, whereas TSA subtype neoantigens are produced *de novo* upon mutational changes of tumor cells (98). These mutations can favor neoantigen tethering to MHCs, produce a new residue on neoantigens increasing TCR recognition, or generate a proteolytic cleavage site, providing better processing for antigen presentation (99). Since TSAs are expressed only in malignant cells, they provide great specificity for T cell cytotoxicity (98). Epigenetic regulation of TAAs in tumor cells can also represent an important target for T cell action (99). In melanoma patients, even tumors with a low mutational burden, but with a high expression of TAAs, which is likely mediated by epigenetic mechanisms, showed considerable response to immunotherapy (100).

Cancers with high mutation rates such as melanoma, bladder cancer, and lung cancer show better response to check point inhibition compared with other types with a lower mutational burden, for instance, PDAC (101–105). Especially tumors with mismatch repair (MMR) deficiency or with more microsatellite instability (MSI) are shown to respond better to immunotherapy (106). As a matter of fact, impairing MMR through genetic inactivation of mutL homolog 1 gene (MLH1) in PDAC mouse models provoked hypermutation, triggering more neoantigen production. This, in turn, prolonged immunosurveillance with better therapeutic response to immune check point inhibitors (Figure 2) (107). Humphris et al. reported that among the 385 resected patient samples only 1% of showed MSI with inactivation of MLH1 and MSH2 (mutS protein homolog 2). This may provide a possible explanation for low response rate to immunotherapy in PDAC (108). Pembrolizumab, a PD-1 antibody, was approved by the Food and Drug Administration in 2017 for solid tumors with MMR defects or MSI, including PDAC (106). Use of DNA damage response (DDR) inhibitors may also enhance the genetic instability of the cancer cells upon exposure to DNA damaging agents, increasing the production of neoantigens. On the other hand, DDR inhibition may show a tumorigenic effect by acting on antitumor immune cells (109). Still, mutational load is not a reliable biomarker for the prediction of response to immunotherapy, considering the patients who were not responding to immunotherapy even if they had a high mutation burden. Likewise, tumors with a low mutational load, such as renal cell carcinomas, responded well to immunotherapy (28, 110).

Recently, Balachandran et al. described a neoantigen quality fitness model identifying long-term survivors of PDAC *via* selecting neoantigens with great resemblance to disease derived peptides (111). On the other hand, a neoantigen quantity model showing more immunosurveillance in response to increasing neoantigen numbers revealed no long-term survivors by itself. Only tumors showing both, high neoantigen numbers and abundant CD8^+^ cytotoxic T cell infiltration, were associated with a significant survival benefit for the patients. Supporting the immune quiescence-like phenotype of PDAC, a modest decrease in high-quality neoantigen transcript levels was seen. More strikingly, they identified a loss of high-quality neoantigen expression in metastatic tumors compared with their primary counterparts. In conclusion, identification of hotspot neoantigens and methods to exploit or target them may increase the response to checkpoint inhibition, not only regarding the primary tumor but also regarding metastatic lesions.

Another mode of reduced antigenicity is the loss of antigen presentation, which can reduce immunosurveillance either by blocking priming of naïve T cells, or by making cancer cells invisible to effector T cell function (97, 112). In other cancer types, reduced antigen presentation was achieved by downregulation of MHC class proteins or impaired antigen processing and shuttling (113–116). Oncogenic RAS signaling was shown to reduce antigen presentation in different cancer types, including PDAC (113, 117, 118). Also, HLA-1 and transporter for antigen presentation production was demonstrated to be reduced in human PDAC specimens (113, 117–119). Manipulating cancer cells for enhanced antigen presentation can reinforce the checkpoint inhibition response.

As a matter of fact, Pommier et al. recently showed that disseminated cancer cells (DCCs, metastatic, quiescent single cancer cells) are undergoing a Darwinian-like selection during immune surveillance of metastasis (120). These investigators elegantly showed that only the metastatic cancer cells, negative for MHC-I and CK-19 expression on the surface, could form DCCs, avoiding T cell-mediated killing in pre-immunized mice. ER stress was the barrier for DCCs to maintain a quiescent state, and also to escape from T-cell-mediated immunity. Therefore, to form macrometastasis, in addition to ER stress relieve, a systemic immunity depletion was required. All these results show how important it is for cancer cells (both primary and metastatic tumor cells) to have good quality neoantigens, and a competency to present neoantigens through MHC complexes to immune cells.

The correlation between total neoantigen load and checkpoint inhibition response is absent in PDAC unlike in other, immunogenic tumors, such as melanoma or lung cancer (104, 121–125). This implies that other factors, determined by the immunogenic properties of PDAC, play an important role in the response to immunotherapy of this malignancy.

### Immunogenicity

Immunogenicity of cancer refers to its ability to induce an adaptive immune response. Based on comprehensive integrated genomic analysis, PDAC was classified into different subgroups by several studies (126–130). RNA expression analysis identified an immunogenic subtype of PDAC in 25 among 96 PDAC patient specimens. This subtype is associated with an increased immune cell infiltration, and enriched signatures such as CD4^+^ and CD8^+^ T cell signaling, antigen presentation, B cell signaling, and most notably CTLA-4 and PD-1 signaling. Signatures enriched in immunogenic subtype might represent predictive biomarkers for immunotherapeutic response in PDAC (130).

Cytolytic activity is determined by the transcription levels of granzyme A (GZMA) and perforin (PRF1), which are known cytotoxicity markers of CD8^+^ T cells (131). Interestingly, genetic amplification of MYC and/or deletion of CDKN2A/B were associated with reduced cytolytic activity in TCGA PDAC datasets (125). Mutant Kras-mediated immunosuppression *via* GM-CSF or IL17R production might be another reason for impaired cytolytic activity in PDAC (132–134). Other than oncogenic drivers, stromal composition may have an impact: PDAC with so-called “normal” stroma (127) (i.e., a good version of stroma, characterized by high ACTA2, VIM, and DES pancreatic stellate cell-PSC markers) was associated with a higher cytolytic activity (125). Considering various factors determine cytolytic activity other than neoantigenic capacity, it is important to adapt individualized precision immunotherapy covering different determinants of immunogenicity in PDAC (125, 135). For an ease of understanding, the determinants of immunogenicity can be divided into two: intrinsic and extrinsic factors.

#### Intrinsic Determinants of Immunogenicity

Antigenic tumors can still evade ICI therapy *via* downregulation of tumor cell-intrinsic immunogenicity (97). In various cancers, stimulation of oncogenic pathways such as PI3K (136, 137), MYC (138), TAZ (139), and JAK-STAT (35) through either excessive ligand production or their mutations induces constitutive PD-L1 production (Figure 2). Myeloid cell induction of EGFR and MAPK signaling in PDAC cells enhanced PD-L1 production inhibiting CD8^+^ T cell infiltration (140). The expression of PD-L1 in various tumors was associated with higher immune cell infiltration and the presence of lymphoid aggregates, and tumors with naturally high levels of PD-L1 in these showed comparably high response rates to anti-PD-1 or anti-PD-L1 (38, 83, 141). Regulation of PD-L1 and other checkpoint inhibitors or oncogenic signaling cascades in cancer cells also constitute an important place for the regulation of tumor immunogenicity (142–144).

Although initial IFNγ production is favorable for CTL activity, chronic exposure may lead to immunoediting in tumor cells. As a result of this, tumors develop genetic or epigenetic modifications in IFNγ signaling components such as IFNγ receptors (IFNGR1 and IFNGR2), JAK-STAT pathway components and IRF1 transcription factors (145, 146). A loss of function mutation on Apelin receptor has recently been identified impairing IFNγ induced JAK-STAT signaling cascade in melanoma (147). Although IFNγ is considered to be antitumorigenic, its induction of PD-L1 transcription in cancer cells might positively correlate to anti-PD-1 or PD-L1 therapy response in established tumors (148). Since IFNγ exposure of cancer cells induces PD-L1 production, mutations in IFNγ signaling components JAK1 and JAK2 would lead to clonal evolution of PD-L1-negative tumor cells, which are not responsive to anti-PD-1 treatment (149). Although no such mutations have been identified in PDAC, personalized medicine can favor the prediction of checkpoint inhibition response through identification of these type of mutations.

Several solid tumors including PDAC showed anti-PD-1 resistance signatures (IPRES) such as enhanced mesenchymal transformation, cell adhesion, extracellular matrix modeling, angiogenesis, hypoxia, and wound healing in TCGA datasets (123). Overall, the differential mutational and transcriptional landscape of tumors does not only determine neoantigen quality and quantity but also regulates several signaling pathways responsible for intrinsic and extrinsic properties of immunogenicity in cancer.

#### Extrinsic Determinants of Immunogenicity: Modulation of TME

Cytotoxic T lymphocyte infiltration into the TME is essential for ICI therapy (150). Even if the anti-tumor CTL infiltration is seen in many tumor types, PDAC represents an outlier in this manner (92). Starting from the premalignant lesions, its microenvironment restricts the cytotoxic T cell infiltration. The cytotoxic T cell function is limited through the actions of immunosuppressive cells in the TME such as cancer-associated fibroblasts (CAFs), myeloid cells, and inhibitory actions of some T cell subsets, albeit they infiltrate in the TME (97, 151). In support of this, strategies eliminating immunosuppressive populations in the TME enhanced CTL infiltration in various cancers (152, 153). To shift the immunosuppressive environment to a non-immune-privileged status, it is important to be aware of the individual components of the TME and to know how to modulate them.

##### Stromal Remodeling

The characteristic abundant desmoplastic stroma of PDAC can be both beneficial and harmful in terms of carcinogenesis. Studies showed that transplantation of PDAC cancer cells with pancreatic stellate cells increased tumorigenic potential and metastasis (154). However, depletion of stroma in preclinical mouse models also revealed further accumulation of T_regs_ in the TME showing the dual nature of stromal compartment (155). In a study performed on human PDAC tissues, the fibrotic reaction did not impair TIL infiltration, rather fibrosis associated collagen-I amount positively correlated with effector T cell presence (156). However, previous studies showed the inhibitory actions of αSMA^+^ CAFs on CD8^+^ CTLs in PDAC TME (157, 158). These results indicated the presence of (?) tumor heterogeneity not only in terms of cancer cells but also stromal compartments of PDAC (159).

One study revealed that depletion of CAFs could actually be employed to increase the immunotherapy response of PDAC: fibroblast activation protein (FAP^+^) CAFs were shown to induce chemokine (C-X-C motif) ligand 12 (CXCL-12) mediating immunosuppression through limiting effector T cell infiltration (158). Targeted inhibitors of FAP^+^ CAFs or CXCL-12 chemokine (C-X-C motif) receptor (CXCR-4) inhibition *via* AMD3100 increased CD3^+^ T cell accumulation and revealed a synergistic effect with anti-PD-L1 therapy in mouse models (158).

Further studies focusing on focal adhesion kinase (FAK) showed FAK inhibition in cancer cells can remodel stroma, inhibiting immunosuppressive TME cells (160). Combination of FAK inhibitor with gemcitabine and anti-PD-1 increased CD8^+^ CTL infiltration, reducing tumor burden and prolonging overall survival (160). Even though single agents targeting FAK inhibition in PDAC showed no objective response in clinic (161–163), trials combining iFAK (vs.-4718) with gemcitabine and anti-PD-1 are ongoing (NCT02758587).

The importance of interleukin 6 (IL-6) signaling in PDAC has been shown by several groups revealing its importance on both carcinogenesis and persistency (164–166). CAFs are also responsible for the production of pro-inflammatory cytokines other than myeloid cells such as IL-6 (167). Unfortunately, clinical trials targeting IL-6 alone demonstrated no benefit (168). However, preclinical studies targeting IL-6 in combination with PD-L1 showed decreased αSMA^+^ stromal cells and increased CD3^+^ lymphocyte infiltration in KPC and Panc02 subcutaneous and orthotopic transplantation models and a survival benefit in the KPC-Brca2 autochthonous mouse model (169).

Hyaluronan, an extracellular matrix component, is a linear glycosaminoglycan in PDAC, associated with multiple markers of aggressiveness of cancer for instance increased cell proliferation, invasion, and metastasis (170). High hyaluronan expression correlates with worse prognosis in PDAC patients (171). Several drugs have been developed to deplete stromal hyaluronan, such as PEGPH20. In preclinical models, hyaluronan depletion *via* PEGPH20 remodeled stroma, decreased interstitial fluid pressure, and increased drug delivery by enhancing micro-vessel permeability (172–174). As PEGPH20 increased delivery of chemotherapeutic agents in PDAC preclinical models, the same was seen for monoclonal antibodies (trastuzumab) in breast cancer (175). With the use of transplanted and autochthonous PDAC mouse models, *Salmonella*-based IDO-1 depletion (176) was also enhanced with (by means of gibi mi?) PEGPH20 treatment (177). Vitamin D receptor (VDR) was identified as a PSC master regulator for dynamic regulation of stromal composition. Treatment with VDR ligand reduced inflammation and enhanced gemcitabine delivery and efficacy in a mode of action similar to hyaluronan depletion (178). Based on these results, hyaluronan depletion or VDR activation appear as promising combination partners of checkpoint inhibitor monoclonal antibodies in clinical trials.

##### Modulation of Immunosuppressive Myeloid Cells

Tumor-associated macrophages (TAMs) differentiate from resident macrophages or mobile inflammatory monocytes (179). TAM polarization can be both beneficial and harmful in terms of carcinogenesis. M1 differentiation of TAMs is known to be antitumorigenic due to their tumoricidal nature *via* releasing pro-inflammatory cytokines. By contrast, the M2 subtype is pro-tumorigenic, since it suppresses immunosurveillance by secreting anti-inflammatory cytokines, e.g., TGF_β_ and IL-10 or by remodeling tumor stroma (180). Consistent with this, expression of M2-related markers such as CD204 and CD163 negatively correlate with patient survival (181, 182). Derived from immature cells of myeloid origin, MDSCs are known for their neoangiogenic and immune-suppressive activities in TME. MDSCs have been shown to inhibit CTL activity by recruiting T_reg_ subset, modulating amino acid reserves in TME, and pushing T cells toward apoptosis *via* ROS production (183). Also, the presence of immunosuppressive cells such as M2 macrophages, T_regs_, and MDSCs in PDAC negatively correlates with overall survival (155, 184–188). Both pro- and antitumorigenic properties of neutrophils in cancer are reported, and their inhibitory action on CTL activity is known to be mediated by various mechanisms (189). Considering the complexity of immune cells in TME and their crosstalk with T cell activity, it is challenging but important to modulate these mechanisms to boost ICI response in cancer.

Cluster of differentiation 40 (CD40) is a member of the tumor necrosis factor receptor superfamily and is expressed on APCs including monocyte subsets, DCs, macrophages, and B cells (190). CD40 signaling is important for licensing APCs (to maximize their capacity to present antigens) followed by cross-priming of CD8^+^ CTL in lymph nodes (191–193). CD40 agonists mediated an enhancement of adaptive antitumor immunity in preclinical mouse models in various cancer types (194–196). By contrast, treatment of KPC mice with CD40 agonist (FGK45) and gemcitabine transiently blocked PDAC development through re-education of tumor-infiltrating macrophages and stromal remodeling, but was not able to invoke an adaptive antitumor immune response (197). On the other hand, subcutaneous transplantation of KPC cancer cells into syngeneic mice revealed that the same treatment strategy induced an adaptive immune response with CD4^+^ and CD8^+^ T cell infiltration. Consequently, the authors used another “two tumor” model, in which intact KPC tumors (cancer cells with intact TME) were transplanted into endogenous tumor-bearing KPC mice. Here, gemcitabine with FGK45 treatment induced a CD4^+^ and CD8^+^ T cell infiltration into subcutaneous tumor but only CD4^+^ infiltration into endogenous tumor. The barrier for CD8^+^ repletion in spontaneous tumors was exceeded through systemic macrophage depletion. Upon deeper analysis, Ly6C^low^ F4/80^+^ macrophages residing in vicinity of PDAC TME were identified as the responsible physical barrier for CTL infiltration (198). In a similar manner, CTL-mediated antitumor immune responses were not seen in clinical trials with CD40 agonists in various cancers even with the addition of gemcitabine to increase tumor immunogenicity (199–202). CD40 agonist treatment finally acted as a checkpoint co-activator through its action on APCs inducing T cell priming upon gemcitabine/nab-paclitaxel dual treatment (203). In conclusion, PDAC retains its antigenic properties to induce both innate and adaptive immune response. This antigenicity might be increased through the use of chemotherapeutics or targeted therapy. Yet, since PDAC is immunologically cold (i.e., very scarce resident CTL infiltration) to respond to increased antigenicity, mechanisms to enhance CTL infiltration must be elucidated. Combination of gemcitabine with nab-paclitaxel remodels TME to permissive conditions for CTL infiltration, but not with gemcitabine alone (203). Furthermore, once T cell priming barrier is exceeded through CD40 agonist treatment, CTL activity might be more expedited with checkpoint inhibitor usage.

C-X-C motif chemokine receptor 2 (interleukin 8 receptor beta, CXCR-2) is a G-protein-coupled receptor for various CXCL ligands including IL-8. CXCR-2 in a cell type-specific manner can act both as a tumor suppressor where it induces senescence in premalignant lesions of PDAC (204, 205) and as tumor promoting *via* enhancing neutrophil and MDSC recruitment to TME (152, 206–208). Through inhibition of CXCR-2 either genetically or pharmacologically with CXCR-2 pepducin (209, 210) or AZ13381758 (211) inhibitors, Steele et al. showed an enhanced response to anti-PD-1 therapy and decreased metastasis in PDAC (212). The enhanced therapy response is reasoned by reduced infiltration of monocytes and MDSCs, which augments T cell infiltration in TME. Also, they propose that stromal remodeling through T cell recruitment might enhance gemcitabine efficacy in tumors (212, 213).

Colony-stimulating factor 1 receptor (CSF1R) is an important regulator of TAMs’ differentiation and sustenance in microenvironment (214, 215). Therefore, inhibition of CSF1R is considered to have potential for cancer therapeutics. Yet, single-agent use targeting CSF1R did not yield clinical benefits in various tumor types (215). In mouse models, treatment of PDAC with CSF1R inhibitors enhanced antitumor immune response; however, this effect was diminished due to the production of checkpoint proteins such as PD-L1 and CTLA-4 (216). Combination of checkpoint inhibitors with CSF1R blockage showed regression of tumors in mouse models (216). CSF1R inhibition was shown to have an effect not only on TAMs but also on CAFs in various subcutaneously transplanted mouse models (217). Recently, CSF1R blockage was shown to enhance the production of granulocyte-specific chemokine expression such as CXCL-1 by CAFs increasing polymorphonuclear MDSC (PMN-MDSC) recruitment as a resistance mechanism (217). PMN-MDSC cells are known for their pro-tumorigenic and anti-immunogenic properties (218). Therefore, combination treatment of CSF1R and CXCR2 inhibitors (see above) targeting, respectively, both TAMs and MDSCs enhanced anti-PD-1 therapy response in transplanted tumor models (217). Considering response enhancement by usage of either CXCR2 or CSF1R inhibitor in combination with immune checkpoint inhibitors, simultaneous use of the three might exploit a broader benefit for therapy response also in PDAC (208, 212).

##### B Cells

Bruton’s tyrosine kinase (BTK) is an enzyme expressed in B cells, macrophages, and mast cells, and targeting BTW in combination with ibrutinib was shown to be effective in chronic lymphocytic leukemia, Mantle cell lymphoma, and Waldenstrom’s macroglobulinemia (219–221). Besides targeting BTK, ibrutinib also inhibits interleukin-2-inducible T-cell kinase in T cells, skewing Th differentiation toward Th_1_ (222). Because of this effect, dual combination of ibrutinib with anti-PD-L1 inhibitor was shown to have a synergistic effect in a T cell-dependent manner, but not MDSC dependently in studies with mouse transplantation models of lymphoma, breast, and colon cancer (219). In various PDAC preclinical mouse models, ibrutinib demonstrated its antitumorigenic effect *via* depletion of macrophage deposition and fibrosis (220). In another study, on the other hand, ibrutinib enhanced macrophage production of Th_1_ differentiation cytokines, while inhibiting Th_2_, and augmenting the CD8^+^ cytotoxic T cell deposition in tumors. The effect on macrophage activity was also dependent on B cells, and B cell-specific BTK signaling, still there was no change in fibrosis (221). Based on these results, checkpoint inhibition in combination with BTK inhibitor ibrutinib might enhance the therapeutic benefit of single use of each in PDAC, accordingly clinical trials are ongoing.

##### γδT Cells (γδT)

T cells are broadly divided into two subtypes based on the antigen receptor types they express: αβT and γδT (223). While 95% of the CD3^+^ T cells in blood express αβTCR (includes CD4^+^ and CD8^+^ T cells) recognizing MHC class I–II, 5% have γδTCR which does not require MHC engagement for activation: γδT are cytolytic through the release of inflammatory cytokines (224, 225). There are conflicting data about the function of γδT in PDAC, with both pro- and antitumorigenic potential. Isolated γδT were shown to be tumoricidal to PDAC cell lines *in vitro* (226). By contrast, in mouse models, pre-neoplastic lesions with KRAS^G12D^ were shown to recruit IL-17-expressing immune cells including γδT, which accelerated carcinogenesis through IL-17 receptor oncogenic signaling (133). In support of this, genetic and therapeutic depletion of γδT in mouse models prolonged survival. Other than the IL-17-mediated oncogenic effect on PanIN lesions, γδT directed checkpoint receptor inhibitory action (through galectin-9 and PD-L1 expression) on αβT cells, accelerating carcinogenesis. While ablation of CD4^+^ and CD8^+^ T cells had no impact on PDAC generation and persistency, this was different upon δTCR knock out: γδT cell deletion increased CD8^+^ CTL and CD4^+^ Th_1_ tumor infiltration, and skewed CD4^+^ differentiation toward the Th1 type. More importantly, the immunosuppressive action of γδT cell was not due to an effect on MDSCs or TAMs; instead, it was directly dependent on checkpoint co-inhibitory receptor engagement with antitumor T cells. PD-L1 and Galectin-9 checkpoint inhibition was effective in tumors with γδT cell present, but not in their absence. This implies the importance of personalized medicine, through which the γδT cell presence may be characterized in patients, to predict checkpoint inhibition therapy response (227).

##### Metabolic Regulation

Enhancing checkpoint inhibition efficiency may also be achieved through regulation of metabolic properties of T cells. For cytotoxic and effector T cell activity, a metabolic switch from a catabolic to anabolic state is important (228–231). While naïve T cells rely mostly on oxidative phosphorylation, activated T cells prefer to switch aerobic glycolysis for faster ATP production (230, 231). In support of this, T cells in anergic state even with TCR engagement and costimulator checkpoint activation can retain their hyporesponsive state in a nutrient poor environment (232). The nutrient poor microenvironment with low glucose and amino acid reservoir is regulated by both cancer cells and the TME (233). Cancer cells, for example, outcompete T cells for glucose uptake having implications for intrinsic immunogenicity regulation (234). Furthermore, glutamine usage by cancer cells also limits its presence in TME, limiting its activator function on T cells (235, 236). ARG-1 (Arginase 1) produced by TAMs and MDSCs degrades arginine (237, 238), while indoleamine 2,3-dioxygenase (IDO-1) produced by cancer cells, TAMs, and MDSCs converts tryptophane to an immunosuppressive metabolite kynurenine reducing T cell activity (239–244). Other than limiting nutrient availability, production of immunosuppressive intermediary metabolic products can also impair T cell activation. Cancer cell production of lactate as a result Warburg effect can impair T cell immunity by both decreasing TME pH and lactate shuttling into T cell (245–247). In addition, adenosine produced by cancer and T_regs_ (248–250), and prostaglandin E2 produced by TAMs and MDSCs are known to inhibit T cell signaling (251). Even though PDAC with its cancer cell and TME components shows similarities in metabolic properties as discussed above, how these metabolic properties effect T cell immunity specifically in PDAC has not been well studied (252). Overall, other than modulation of cytokine–chemokine–receptor axis, nutrient availability and production of immunosuppressive metabolites might also affect the extent of T cell immunity.

The impact of metabolism on checkpoint inhibition efficacy may not be only relevant on a micro environmental but also on a more systemic level. Cachexia is a systemic disorder with an excessive weight loss through the consumption of muscles and adipose tissues (253). Many diseases are associated with cachexia, including cancer in general and PDAC in particular (253). An increase in serum IL-6 levels was shown to impair hepatic ketogenesis inducing cachexia in C26 colon cancer and autochthonous KPC-PDAC mouse models (254–256). Physiologically, the body responded to cachexia with an upregulation of glucocorticoids like (?) corticosterone, which inhibits T cell infiltration into tumors of C26 cells. In support of this, transcriptomics analysis of pre-cachectic and cachectic C26 transplanted mice revealed an impaired immunological phenotype. However, this signature was not seen in the KPC model of PDAC, again implying its innate immunocompromised nature (254). With the use of the CXCR-4 inhibitor AMD3100, this barrier was overcome, increasing T cell infiltration and PD-L1 checkpoint inhibitor efficiency (158, 254). However, with the addition of corticosterone to the AMD3100-PD-L1 combination, the therapeutic effect was diminished (254). These results have multiple implications for PDAC therapeutics: the checkpoint inhibition resistance might be tackled with glucocorticoid synthesis inhibition, though this might first require a prior consideration for CTL infiltration. Second, serum glucocorticoid levels might be important markers for checkpoint inhibition response in PDAC patients. And finally, serum IL-6 depletion might provide further opportunities to increase the checkpoint inhibition efficacy, not only because of its direct effect in the TME but also due to its physiological role in cancer-related cachexia (254).

## Other Combination Strategies Exploiting Antigenicity/Immunogenicity of Tumors to Enhance Checkpoint Inhibition Therapy

**Figure 4:**
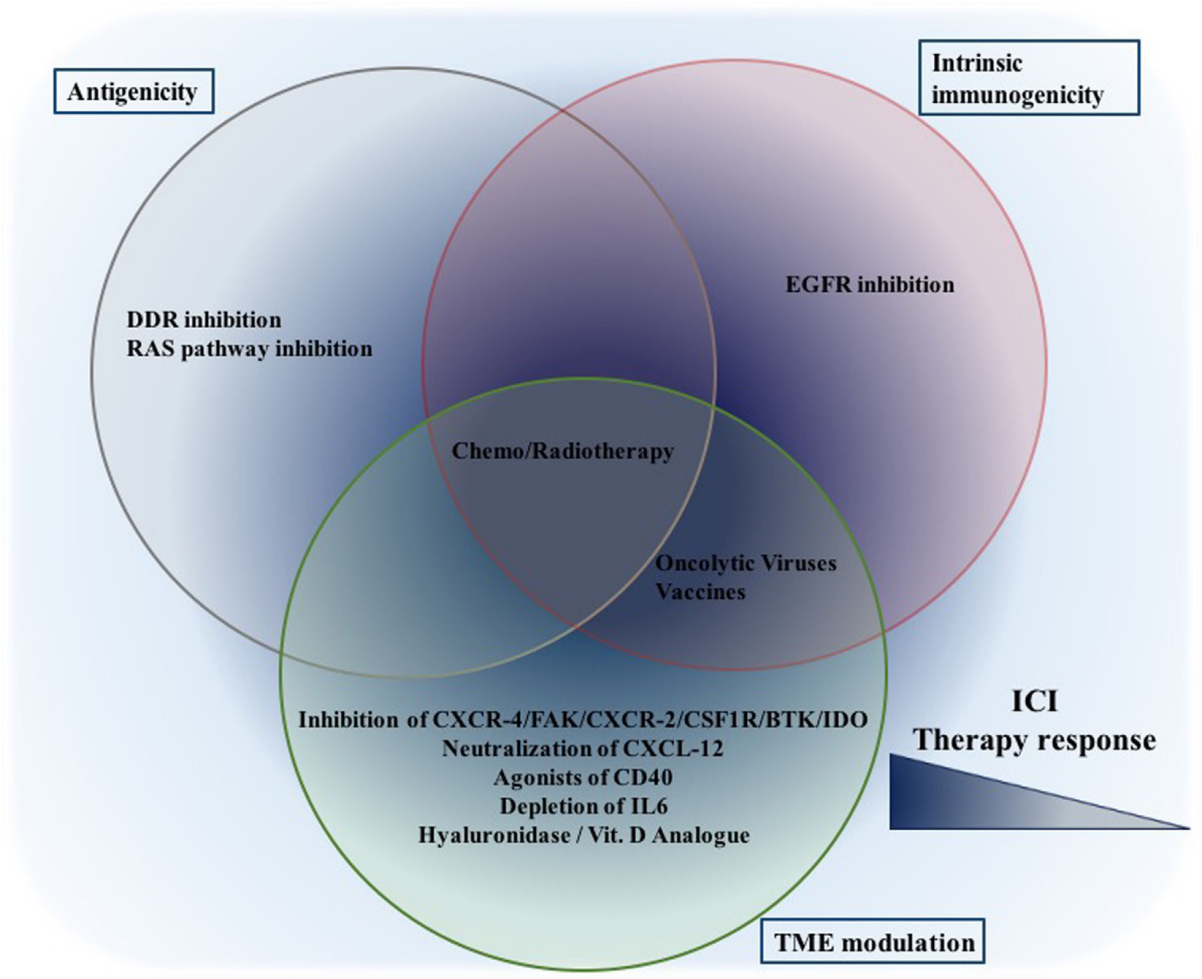
Combination therapeutic options to increase ICI efficiency: while given therapeutic options are placed in the corresponding cluster, only published data thus far are taken into consideration. This still does not eliminate their potential to affect other aspects. While treatments focusing on a single aspect (either one of antigenicity/intrinsic immunogenicity/TME modulation) might be effective, the best synergism will probably be achieved through combinations focusing on all aspects. Abbreviations: ICI, immune checkpoint inhibition, DDR, DNA damage response.

Approaches including specific inhibitors (small molecules and antibodies) of various signaling pathways are described thus far and listed in Table 2. Apparently, combining the classical, untargeted treatment strategies, chemotherapy and RT, with checkpoint inhibition in clinical trials appears reasonable. Other targeted immunotherapeutic options, e.g., oncolytic viruses, vaccines, and chimeric antigen receptor-T cell (CAR-T) therapies aim to treat cancer in a more specific manner with minimal side effects. Selected clinical trials combining immune checkpoint inhibitors with untargeted and other targeted immunotherapeutic options are listed in Table 3. Combination therapies can modulate both antigenic and immunogenic landscape of tumors (Figure 4). Likely more important than just developing novel combination partners, exact understanding of the mode of action of combination partners, their tolerability and toxicity, a determination of dosing and appropriate sequencing of the combinations are required (257).

**Table 2:** Selection of currently ongoing clinical trials evaluating CTLA4 or/and PD1/PD-L1 checkpoint blockade in combination with targeted therapy approaches for pancreatic cancer as indicated.

Combination strategy/target	Compounds	Entity	Phase	Trial ID
Oncogenic signaling	Cobimetinib (MEK-inh.) + atezolizumab (PD-L1-Ab)	Metastatic PDAC, progressed on chemotherapy	Ib/II	NCT03193190
TME: stroma	Ulocuplumab (CXCR-4-ant.) + nivolumab (PD-1-Ab)	Advanced/metastatic pancreatic cancer (next to SCLC)	I/II	NCT02472977 (terminated 03/2018 due to lack of effic. in short-term ph.)
	BL-8040 (CXCR4-ant.) + pembrolizumab (PD-1-Ab)	(Pretreated) metastatic pancreatic cancer	II	NCT02826486 and NCT02907099
	BL-8040 (CXCR4-ant.) + atezolizumab (PD-L1-Ab)	Metastatic PDAC, progressed on chemotherapy	Ib/II	NCT03193190
	Olaptesed pegol (pegylated oligoribonucleotide, neutralizing CXCL12) ± pembrolizumab (PD-1-Ab)	Metastatic pancreatic cancer (next to CRC)	I/II	NCT03168139
	Defactinib (FAK-inh.) + pembrolizumab (PD-1-Ab)	Advanced pancreatic cancer (next to NSCLC and mesothelioma)	I/II	NCT02758587
	PEGPH20 (pegylated recombinant human hyaluronidase) + atezolizumab (PD-L1-Ab)	Metastatic PDAC, progressed on chemotherapy	I/II	NCT03193190
	PEGPH20 (see above) + avelumab (PD-L1-Ab)	Chemotherapy resistant advanced pancreatic cancer	I	NCT03481920
	Pembrolizumab (PD-1-Ab) ± paricalcitol (vitamin D analog)	Maintenance of pretreated advanced pancreatic cancer in (partial) remission	II	NCT03331562
TME: myeloid	RO7009789 (CD40 ago. Ab) + atezolizumab (PD-L1-Ab)	Locally advanced/metastatic solid tumors	I	NCT02304393
	Cabiralizumab (CSF1R-Ab) + nivolumab (PD-1-Ab)	Advanced solid tumors	I	NCT02526017
	AMG820 (CSF1R-Ab) + pembrolizumab (PD-1-Ab)	Advanced pancreatic cancer (next to CRC and NSCLC)	I/II	NCT02713529
	Pedixartinib (CSF1R-tyrosine kinase inh.) + durvalumab (PD-L1-Ab)	Pretreated advanced/metastatic pancreatic cancer (next to CRC)	I	NCT02777710
	Acalabrutinib (bruton tyrosine kinase inh.) + pembrolizumab (PD-1-Ab)	Metastatic pancreatic cancer	II	NCT02362048
TME: metabolism	Epacadostat (IDO1-inh.) + pembrolizumab (PD-1-Ab)	Previously treated advanced pancreatic cancer (with chromosomal instability/HRRD)	II-withdrawn	NCT03432676

*Ab, antibody; inh., inhibitor; ant., antagonist; ago., agonist; CRC, colorectal cancer; HRRD, homologous recombination repair deficiency; IDO1, indoleamine 2,3-dioxygenase 1; NSCLC, non-small cell lung cancer; SCLC, small cell lung cancer; TME, tumor microenvironment; PD-1, programmed cell death protein 1*.

**Table 3:** Selection of currently ongoing clinical trials evaluating CTLA4 or/and PD1/PD-L1 checkpoint blockade in combination with untargeted and targeted options including other immunotherapeutic approaches for pancreatic cancer as indicated.

Combination strategy/target	Compounds	Entity	Phase	Trial ID
Chemotherapy	Gemcitabine + ipilimumab (CTLA-4-Ab)	Advanced pancreatic cancer	Ib	NCT01473940
	Nab-paclitaxel (±gemcitabine) + nivolumab (PD-1-Ab)	Advanced/metastatic pancreatic adenocarcinoma (next to NSCLC and mBC)	I	NCT02309177
	mFOLFOX6 + pembrolizumab (PD-1-Ab) [+celecoxib (COX-2-inh.) for non-responders]	Advanced gastrointestinal-cancer including pancreatic cancer	I	NCT02268825
Radiotherapy	SBRT 6 Gy × 5 days + durvalumab (PD-L1-Ab), vs. tremelimumab (CTLA-4-Ab) vs. both combined	Unresectable, non-metastatic pancreatic cancer	Ib	NCT02868632
	SBRT 5 Gy × 5 days vs. 8 Gy × 1 day + durvalumab (PD-L1-Ab), vs. tremelimumab (CTLA-4-Ab) vs. both combined	Unresectable pancreatic cancer	I/II	NCT02311361
	Radiotherapy (not defined) + nivolumab (PD-1-Ab) and ipilimumab (CTLA-4-Ab)	Pancreatic cancer, progressed on chemotherapy (next to CRC)	II	NCT03104439
	45–50.4 Gy + PD-1-Ab (not defined)	Unresectable pancreatic cancer	II	NCT03374293
Vaccines	GVAX/Cy ± nivolumab (PD-1-Ab)	Neoadjuvant/adjuvant for resectable pancreatic cancer	I/II	NCT02451982
	GVAX/Cy + CRS-207 ± nivolumab (PD-1-Ab)	Previously treated metastatic pancreatic adenocarcinoma	II	NCT02243371
	CRS-207 (±GVAX/Cy) + nivolumab (PD-1-Ab) and ipilimumab (CTLA-4-Ab)	Previously treated pancreatic cancer	II	NCT03190265
Chemotherapy + vaccine	Capecitabine + CV301 + durvalumab (PD-L1-Ab)	Metastatic pancreatic cancer (next to CRC)	I/II	NCT03376659
Chemotherapy + Vit. D analog	Paricalcitol (vitamin D analog) + pembrolizumab (PD-1-Ab) ± gemcitabine/nab-paclitaxel	Resectable pancreatic cancer, neoadjuvant setting	I	NCT02930902
Chemotherapy + FAK	Defactinib (FAK-inh.) + gemcitabine + pembrolizumab (PD-1-Ab)	Advanced solid tumors	I	NCT02546531
Chemotherapy + CD40	Gemcitabine/nab-paclitaxel + APX005M (CD40-ago.-Ab) ± nivolumab (PD-1-Ab)	Untreated metastatic pancreatic adenocarcinoma	II	NCT03214250
Chemotherapy + CSF1R	Cabiralizumab (CSF1R-Ab) + nivolumab (PD-1-Ab) ± different chemotherapeutic regimens	Pretreated, progressed metastatic pancreatic adenocarcinoma	II	NCT03336216
Radiotherapy + vaccine	SBRT 6.6 Gy × 5 days + GVAX/Cy + nivolumab (PD-1-Ab)	Borderline resectable pancreatic cancer, no previous therapy	II	NCT03161379
Radiotherapy + vaccine	SBRT 6.6 Gy × 5 days + GVAX/Cy + pembrolizumab (PD-1-Ab)	Locally advanced pancreatic cancer	II	NCT02648282
CSF1R + vaccine	IMC-CS4 (CSF1R-Ab) + GVAX/Cy + pembrolizumab (PD-1-Ab)	Borderline resectable pancreatic adenocarcinoma	I	NCT03153410
IDO1 + vaccine	Epacadostat (IDO1-inh.) + CRS-207 (±GVAX/Cy) + pembrolizumab (PD-1-Ab)	Metastatic pancreatic cancer progressed on prior chemotherapy	II	NCT03006302
ACT	Autologous TIL, ipilimumab (CTLA-4-Ab), nivolumab (PD-1-Ab), proleukin, Cy., fludara	Cancer patients across all diagnoses	I/II	NCT03296137

*Ab, antibody; inh., inhibitor; ago., agonist; CRC, colorectal cancer; CRS-207, *Listeria*-based mesothelin vaccine; CV301, CEA/MUC1 prime-boost vaccine based on modified vaccinia Ankara-Bavarian Nordic (MVA-BN), a recombinant fowlpox viral vector (foe the boost) and TRICOM, which is comprised of three costimulatory molecules B7-1, ICAM-1, and LFA-3; Cy, cyclophosphamide; GVAX, irradiated pancreatic cancer cells, genetically modified to express GM-CSF; IDO1, indoleamine 2,3-dioxygenase 1; mBC, metastatic breast cancer; NSCLC, non-small cell lung cancer; SBRT, stereotactic body radiation therapy; ACT, adoptive cell therapy; TIL, tumor-infiltrating lymphocyte; CTLA-4, cytotoxic T lymphocyte-associated antigen 4; PD-1, programmed cell death protein 1*.

### Combination of Immune Checkpoint Inhibitors With Untargeted Therapeutic Options

#### Chemotherapy

The first-line PDAC therapeutics used in clinic are chemotherapeutic agents such as gemcitabine with/without nab-paclitaxel, and FOLFIRINOX (folinic acid, fluorouracil, irinotecan, and oxaliplatin) (258). These agents are known for their ability to induce cytotoxicity due to impaired cell division. The mutagenic effect of chemotherapy (or RT) may enhance neoantigen production and MHC class I antigen presentation on cancer cells, increasing tumor antigenicity (109). Still, even if sub-clones with reactive neoantigenic properties might evolve, they might not be substantial enough to result in a broad clonal response in response to checkpoint inhibition (109). Furthermore, considering that PDAC already retains its antigenic capacity but its immunosuppressive microenvironment is the main barrier to pass as explained above, chemotherapy might exert its effect rather by altering immunogenicity. Immunogenic cell death (ICD) upon chemotherapy releases danger signals and cytokines for the generation of a more immunogenic TME (109). As also seen in the gemcitabine with nab-paclitaxel example above, remodeling of the immunosuppressive TME can bolster up T cell cytotoxicity due to enhanced immunogenicity. Chemotherapy can increase immunogenicity by also its direct action on immunosuppressive cells of TME. For example, fluorouracil and paclitaxel were shown to induce MDSC apoptosis in various tumor models, while low dose gemcitabine was shown to deplete T_regs_ in panc02 orthotopic mouse model (259). Furthermore, it will be important to select chemotherapeutic agents, their dosing and time and sequence of administration with regard to their ability to induce ICD and remodel the microenvironment.

#### Radiotherapy

Although the use of RT for the treatment of PDAC has been controversially discussed due to rather disappointing results in clinical trials (260), radiation treatment in combination with ICI might be a promising strategy for pancreatic cancer patients. In a phenomenon known as the abscopal response, RT was shown to induce immune responses that mediate regression of metastatic lesions lying outside the field of radiation (261). RT could activate the immune system, increase trafficking of T cells to the tumor, and elicit antitumor immune responses following ICD (262). Several preclinical and clinical studies in different cancer types showed synergistic effects in cohorts treated with RT and immune checkpoint blockade (263–266). Although not many studies have been published thus far, evidence for synergism can also be seen in PDAC and has been related to increased immunogenicity (95, 267, 268). In the PDAC mouse model used by Twyman-Saint Victor et al., any combination of immune checkpoint inhibitor with RT substantially increased overall survival, compared with immune checkpoint blockade with either CTLA-4 antibody or PD-1 antibody alone. The highest response rate and longest overall survival was seen in the triple combination therapy (two checkpoint inhibitors + RT) group (269).

Recently, CD40 agonist treatment was demonstrated to be beneficial upon a RT + ICI regimen in murine pancreatic cancer models (270). While RT alone or in combination with ICI resulted in reduction of irradiated tumor growth, only the triple therapy, RT + αCD40 + ICI (RCP4), affected the growth of both irradiated and unirradiated tumors. These observations were also reflected in the long-term survival. Furthermore, CD4 and CD8 T cells, as well as short-lived myeloid cells were shown to be necessary for optimal response to RCP4 and that RCP4 antitumor immunity. This immunity was dependent on host CD40, Batf3, and IFNγ but not on B cells and canonical innate immune activation pathways. The three therapies all showed non-redundant impact on the antitumor immune response. While RT triggered an early pro-inflammatory stimulus, αCD40 caused systemic myeloid compartment reorganization and ICI increases intratumoral T cell infiltration, thus improving the CD8/T_reg_ cell ratio.

In conclusion, RT can enhance the “visibility” of tumor antigens and make the tumor more immunogenic. While the combination of RT and ICI shows promise in preclinical and clinical trials in various cancer entities, challenges still exist for the safe and efficacious application of the combination. Tumor-type and immune therapy-specific optimization of radiation dose and timing and the identification of potential biomarkers is likely to further enhance the effectiveness (271). Also, the addition of αCD40 agonists appears to be a promising avenue to pursue in clinical PDAC trials.

### Combination of Immune Checkpoint Inhibitors With Other Immunotherapeutic Approaches

#### Oncolytic Viruses

Tumor-targeted oncolytic viruses (TOVs) are viruses that selectively infect, replicate in, and lyse tumor cells, while leaving healthy, normal tissues unharmed. TOVs can have intrinsic tumor-selectivity, making them naturally nonpathogenic to humans and sensitive to antiviral signaling (272) or depend on oncogenic signaling pathways, e.g., constitutively activated RAS (273, 274). Viral tumor specificity can also be genetically engineered by deleting genes required for replication in normal tissues (273) or by placing viral replication under the control of a tumor-specific promoter (274–276), TOVs can also be designed to express tumor-specific cell surface receptors (277, 278). TOVs can thus be engineered to increase safety, efficacy, and tissue tropism.

The advantages of TOVs are their specificity, modest toxicity, low probability for resistance, and most importantly, their induction of an inflammatory cascade and engagement of the adaptive immune system (273). In contrast to any other drug, the therapeutic dose of TOVs increases over time, as the virus replicates and spreads to neighboring cells (273). Although TOVs directly lyse infected malignant cells, causing acute tumor debulking, it is the ability of the virus to spread from cell to cell and potentiate an inflammatory response through ICD that make oncolytic viruses such promising new therapies (279–281). However, oncolytic virus therapy faces challenges in solid tumors and especially PDAC. These challenges, i.e., overcoming the TME, avoiding neutralization by the host immune system, and acquired resistance in tumor cells culminate in the main problem, i.e., the systemic delivery of TOVs for the targeting of metastatic cancer cells (282). Thus, it is not surprising that thus far, no studies investigating ICI and oncolytic viral therapy in pancreatic cancer have been published. However, Mahalingam et al. (283) conducted a phase II study of pelareorep, a proprietary replication-competent isolate of reovirus type 3 dearing in combination with gemcitabine in advanced PDAC and observed the upregulation of PD-L1 in following treatment. They suggested to investigate the combination of oncolytic virus therapy with anti-PD-L1 inhibitors in PDAC.

Congruent with this finding, recent research in other cancer entities revealed that antiviral immunological events induced by the administration of oncolytic viruses can turn tumors “hot” (284, 285) and establish a TME that is conducive for enhancing the efficacy of checkpoint inhibitors (286–288). Using intravenous infusion of oncolytic human orthoreovirus, Samson et al. (288) found that TOV treatment increases cytotoxic T cell tumor infiltration, upregulates IFN-regulated gene expression, and the PD-1/PD-L1 axis in tumors, *via* an IFN-mediated mechanism. And finally, addition of PD-1 blockade to reovirus treatment enhanced systemic therapy in a preclinical glioma model. In their simultaneously published triple-negative breast cancer (TNBC) study, Bourgeois-Daigneault et al. (286) reported that TOV therapy sensitizes otherwise refractory TNBC to immune checkpoint blockade, preventing relapse in most of the treated animals.

In conclusion, once the problem of systemic delivery is solved, oncolytic viruses are not only valuable therapies in terms of tumor debulking but are also useful in a “prime and boost” approach in combination with ICIs.

#### Vaccines

Another promising approach to enhance the immunogenicity of pancreatic cancer cells and boost the antitumor T cell response is the use of cancer vaccines. Vaccines have been designed to generate a humoral/cellular immune response with the aim of stimulating the host immune system to recognize and eliminate tumor cells with specific effector and memory T cells. There are two major categories of tumor vaccines: whole cell vaccines and antigen-specific vaccines (289). A brief review on the different vaccines currently investigated for pancreatic cancer can be found in the publication by Skelton et al. (290). Although early studies using single-agent tumor vaccines against PDAC showed improved immune profiles, they were largely unable to produce a positive clinical response (291). This can be explained by the upregulation of immunosuppressive signaling, as well as other immune modulating mechanisms, which negate the positive effects of the vaccine (267, 292).

The induction of T cell infiltration and PD-L1 expression in the TME by vaccine treatment was hypothesized to prime PDACs for anti-PD-1/PD-L1 therapies. Indeed, the whole cell vaccine GVAX, consisting of two allogeneic irradiated PDAC cell lines engineered to secrete GM-CSF, converted a non-immunogenic or “cold” neoplasm into an immunogenic or “hot” neoplasm by inducing infiltration of T cells and development of tertiary lymphoid structures (267, 285). In a subsequent phase Ib study, Le et al. (293) were able to show that the combination of GVAX with ipilimumab induced objective responses in patients with metastatic PDAC that were not observed with either single therapy alone.

Preclinical data suggested beneficial effects when two vaccination treatments were co-implemented, e.g., GVAX and CRS-207, a live-attenuated *Listeria monocytogenes* vaccine expressing the TAA mesothelin, in a sequential combination—a so-called prime/boost approach. The first vaccine was given to initiate or “prime” the immune system, and this immune response was then “boosted” following re-administration of antigen resulting in the induction of a synergistic enhancement of T cell induction and antitumor effect (289). Based on the preclinical data, a phase II trial (NCT01417000) was conducted resulting in the conclusion that heterologous prime/boost with Cy/GVAX and CRS-207 extended the survival of patients with pancreatic cancer, with minimal toxicity (294). Unfortunately, a subsequent phase IIb trial of CRS-207 and GVAX (NCT02004262) did not show a significant difference in overall survival between the groups treated with either CRS-207/GVAX or CRS207 alone and the group treated with chemotherapy, i.e., physicians’ choice of therapies including: gemcitabine, capecitabine, fluorouracil, leucovorin, irinotecan, and erlotinib.

Although the last mentioned study was quite a set-back, the strategy of combining different immunotherapy options with each other still holds a merit, especially for “prime and boost” approach.

#### Chimeric Antigen Receptor (CAR)—T Cell Therapy

Chimeric antigen receptors are fusion proteins that can be comprised of three major domains. These are the antigen-specific ectodomain, commonly derived from a single-chain variable antibody-fragment (scFv); a transmembrane domain fused to a spacer that links to the ectodomain; and an endodomain consisting of different cytoplasmic proteins responsible for T cell activation (295). Unlike endogenous TCRs, CARs recognize their target antigen in an MHC (or HLA)-independent manner, due to their engineered antibody fragment. Upon antigen recognition, CAR-T cells are activated, leading to cytokine secretion, T cell proliferation, and antigen-specific cytotoxicity (296). The production of CAR-T cells for adoptive T cell transfer requires the isolation, stimulation, expansion, transduction, i.e., viral vector-mediated insertion of specific CAR genes, and ultimately reinfusion of autologous or allogeneic T cells (297–299).

Although impressive clinical activities of CAR-T cells in hematological malignancies were reported, CAR T-cell trials in solid tumors have yet to yield the same level of success (300, 301). The most prominent obstacles standing in the way of successful CAR-T cell therapy are (1) lack of ideal TSAs, (2) inefficient trafficking of CAR-T cells to tumor sites, (3) the immune-suppressive TME, and (4) the risk of developing on-target/off-tumor toxicities, i.e., the attack of normal cells expressing the targeted tumor antigen (296).

While the investigation of CAR-T cells in pancreatic cancer is still in early stages, it is fair to say that the first above mentioned obstacle does not apply. PDAC exhibits a number of TSAs and, conceptually, is a promising candidate tumor for investigating CAR T-cell therapy. Thus far, there have been preclinical studies on various pancreatic cancer cell surface antigens, namely, MSLN, CEA, MUC1, PSCA, CD24, HER2, and natural killer receptors (302).

The main obstacles in pancreatic cancer are most likely the strong immunosuppressive TME, already discussed in this review, and improper homing and inefficient infiltration of CAR T-cells to the tumor bed. Especially challenging is the high number of infiltrating T_regs_ and MDSCs, which can deactivate CAR-T cells through cytokines inhibitory cytokines such as TGF_β_ and IL-10, and the upregulation of inhibitory receptors, e.g., PD-1 on adoptively transferred CAR-T cells after homing to the tumor (95, 302–304). T cell hypofunction was reversed when the cells were isolated from the tumor, or after treatment with a blocking PD-1 antibody (304–306), and there are promising preclinical studies on CAR-T cells engineered to secrete PD-1 checkpoint inhibitors (307, 308) or PD-1 dominant negative receptor (304).

These results provide rationale for combination therapies, with CAR-T cells and checkpoint blockade, as a new strategy to overcome the tumor escape and to further strengthen CAR-T cells, especially in patients with PDAC shown to express high levels of PD-L1.

#### Adoptive Cell Therapy (ACT) With Endogenous TILs

Adoptive cell therapy using endogenous TILs taken from surgically resected tumors, expanded *in vitro*, and re-infused back into the patient, is a promising approach for otherwise untreatable cancer types (309). In metastatic melanoma patients, for example, TIL-ACT was associated with a 20% complete response lasting beyond 3 years (310). Gastrointestinal tumor patients with CD3^+^ T cell infiltration showed a higher rate of progression-free survival (311), and pancreatic adenocarcinomas containing both CD4^+^ and CD8^+^ T cells correlated with an improved prognosis and significantly greater 5-year survival (181, 312, 313). This evidence of a host T cell immune response in patients with pancreatic adenocarcinoma drove both Hall et al. (309) and Poschke et al. (314) to expand and analyze the T cell repertoire in resected primary PDAC specimen. Contrary to the common description of PDAC as an immunologically “cold” tumor, they found that most resectable PDA tumors actually contained significant numbers of T-cells and, along with that, tertiary lymphoid structures in which clonal T-cell expansion takes place and were able to expand them *in vitro* using high levels of IL-2 (309, 314). The majority of these TILs were CD4^+^ T cells and were highly activated and resembled those extracted from melanoma samples. Media supplemented with anti-4-1BB significantly increased the TIL yield per fragment and shifted the T cell population to predominantly CD8^+^ cells compared with control cultures. The population of 4-1BB positive CD8^+^ lymphocytes represented the population of tumor-resident TILs specific for expressed tumor antigens on the surface of pancreatic adenocarcinoma cells (309, 314).

Thus far, there has been no studies investigating the combination of TIL ACT and ICI in pancreatic cancer. However, pretreating PDAC patients with immune checkpoint inhibitors and thus enriching the population of tumor-specific lymphocytes prior to surgical resection might be a worthwhile strategy. This way the yield of tumor reactive cells could be increased, the expansion time and the time between surgery and infusion shortened, and thus the risk of recurrent growth during the expansion period decreased.

This is exactly the approach taken by Mullinax et al. when analyzing the combination of TIL ACT and Ipilimumab in a clinical pilot study (NCT01701674) for metastatic melanoma, and in clinical trials for metastatic ovarian cancer (NCT03287674). The pretreatment with Ipilimumab followed by ACT in metastatic melanoma patients was reported as feasible, well tolerated, and associated with a low rate of attrition due to progression during cell expansion (315). The investigators are currently recruiting patients for a similar trial in metastatic melanoma, now including 4-1BB (NCT02652455), and another study with a similar design is currently recruiting patients with locally advanced or metastatic cancers of various types (NCT03296137).

Considering the positive results in metastatic melanoma, as well as the similarity in the population of extractable T cells from melanoma and PDAC, the investigation of ICI + TIL ACT in PDAC is recommendable.

## Concluding Remarks

The revolution of immunotherapy is changing our perspective in cancer therapeutics. For some solid tumors, immunotherapy has already entered into clinical practice. While PDAC is unresponsive and refractory to many of the conventional therapies, immunotherapy holds a promise for future improvement. However, single-agent ICI has largely failed. Based on the findings thus far, the decisive drawback for ICI efficiency in PDAC is the initial T cell priming. Only less than 1% of human PDAC samples are projected to show aberrant genomic instability, enabling T cell priming despite the immunosuppressive microenvironment. However, this does not mean the other 99% are not antigenic, rather its antigenic strength likely cannot beat reduced immunogenicity. Each patient, each tumor, and each cancer cell are distinct. T cells might provide the best repertoire for the recognition of each single difference, yet to overcome immunogenic obstacles, combination strategies are required. Development of the best combinations comes along with better characterization of the patient samples. Characterization of these samples might help us to better classify the individual distinctions that patients, tumors, and cancer cells have, and to find the best combination partners with checkpoint inhibition. Even though complete regression of the primary tumor might not be achieved, reduction and control of metastasis can still provide a considerable prognostic value in PDAC patients. While we know that metastatic lesions evade the expression of high quality neoantigens of their cognate primary tumor and antigen presenting machinery, they might still retain their unique antigenic and immunogenic master regulators to be targeted. Most importantly, T cell memory provides the best tool to minimize disease recurrence, therefore strategies exploiting T cell memory may provide long-term disease control. Before achieving T cell memory, to make PDAC responsive to first time checkpoint inhibition, we have to elucidate and exploit the mechanisms discussed above: (1) increasing initial T cell priming, (2) exceeding immunosuppressive TME, and (3) inhibiting compensatory mechanisms of T cell anergy and exhaustion.

## Author Contributions

DK wrote the manuscript, prepared the figures and tables. KC wrote and edited the manuscript. DR provided the tables and edited the manuscript. HA edited and supervised the manuscript.

## Conflict of Interest Statement

The authors declare that the research was conducted in the absence of any commercial or financial relationships that could be construed as a potential conflict of interest.
